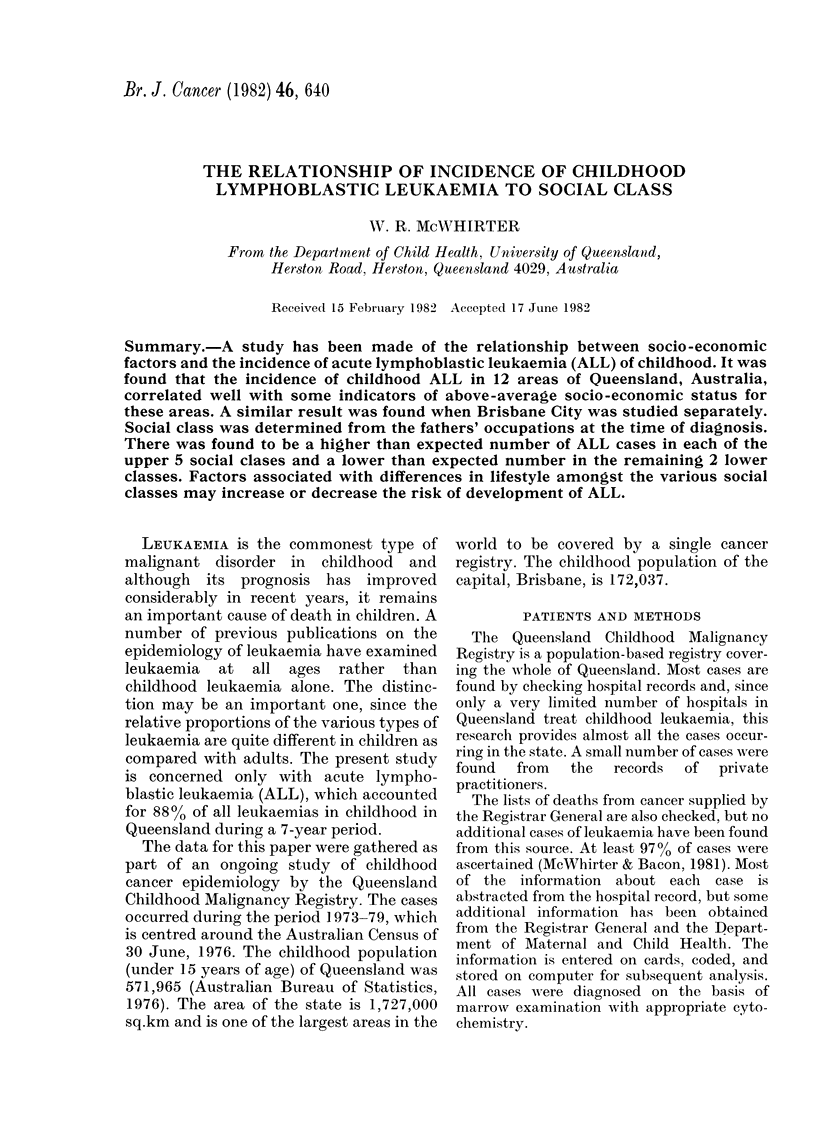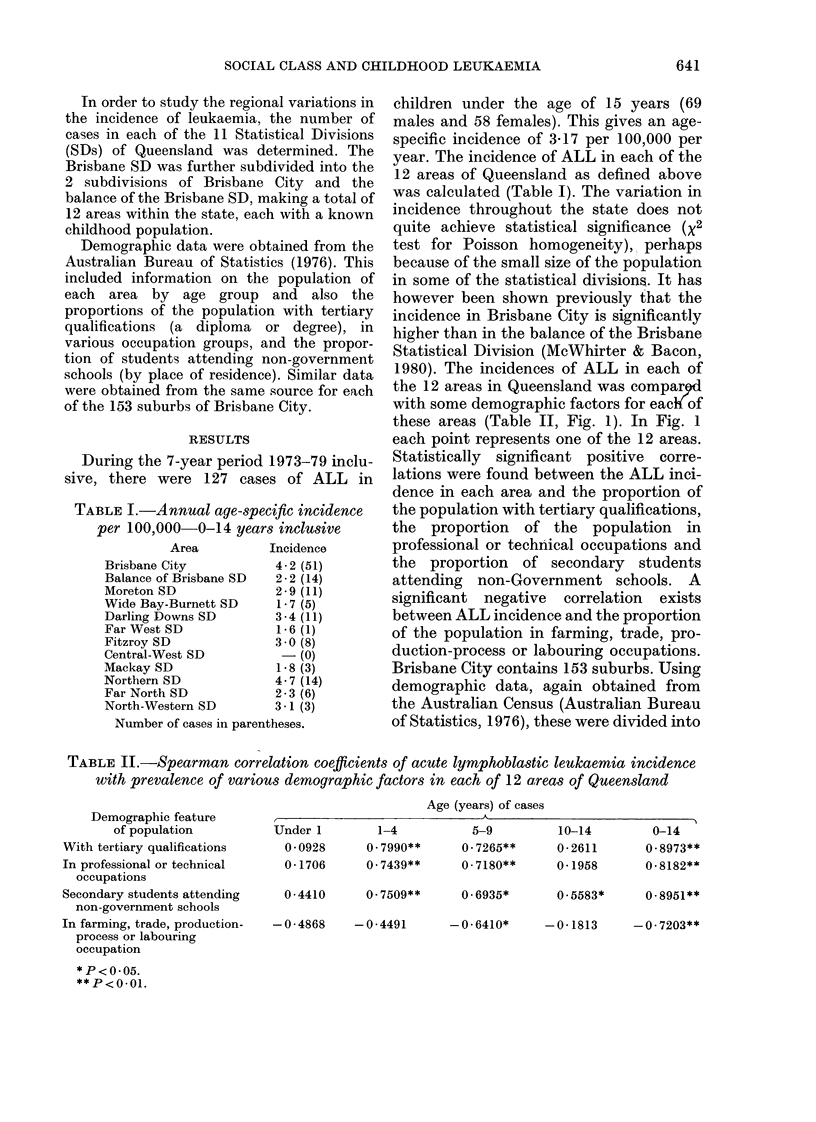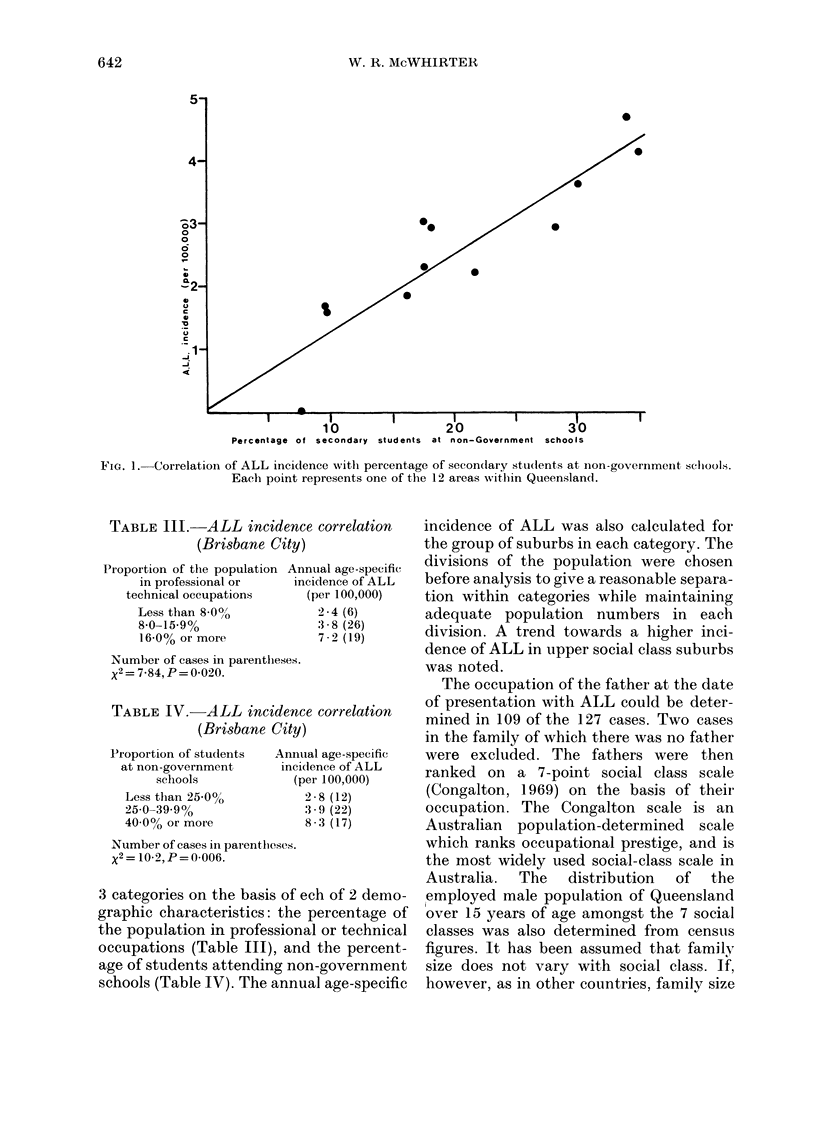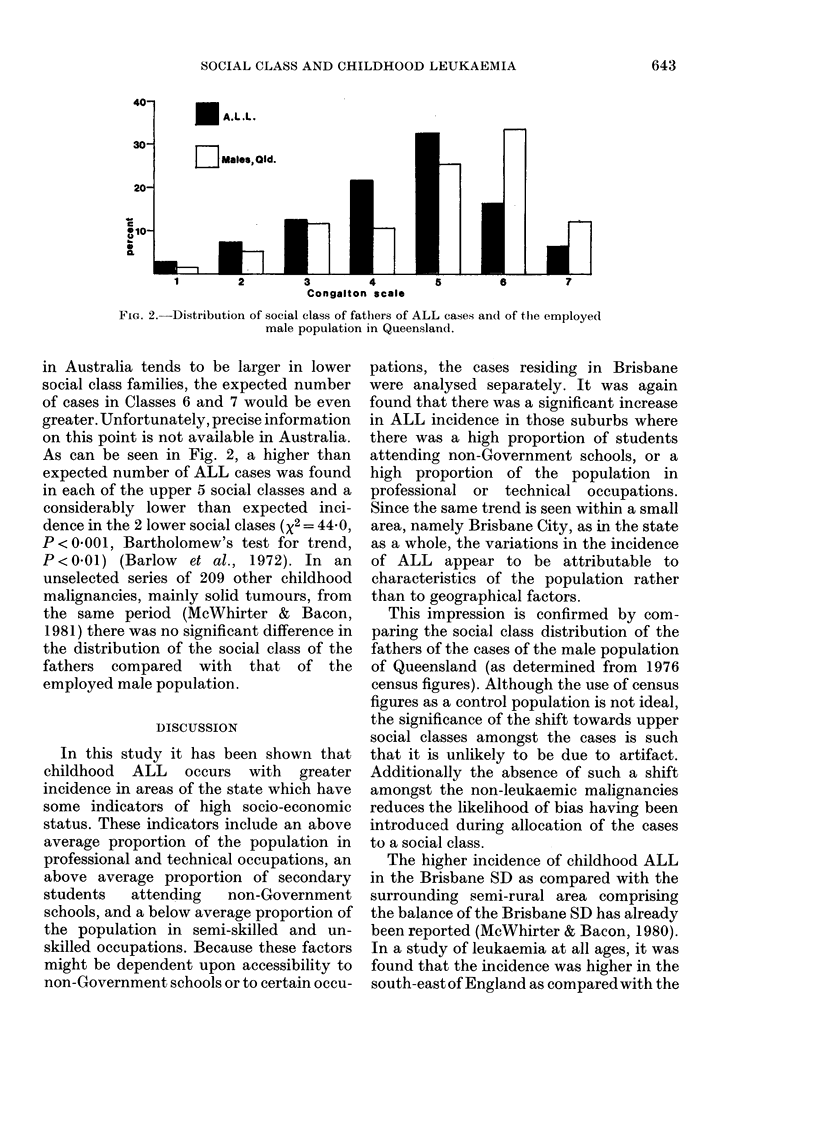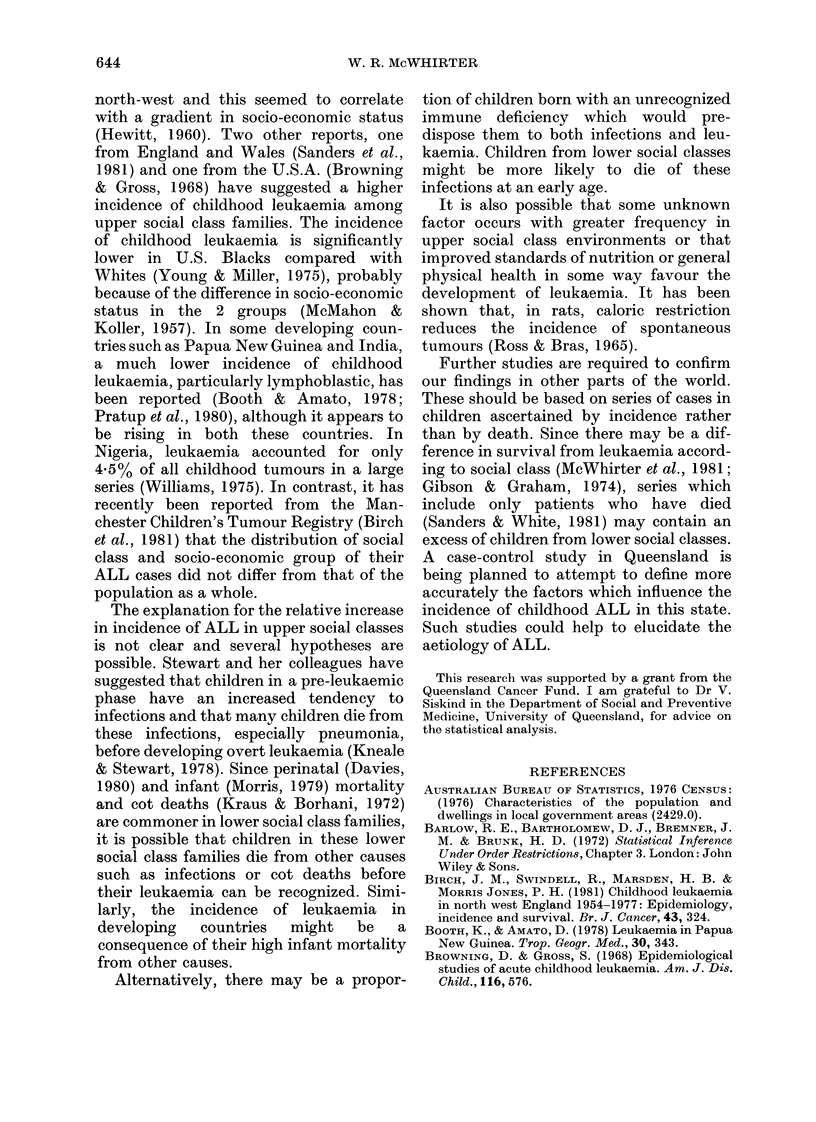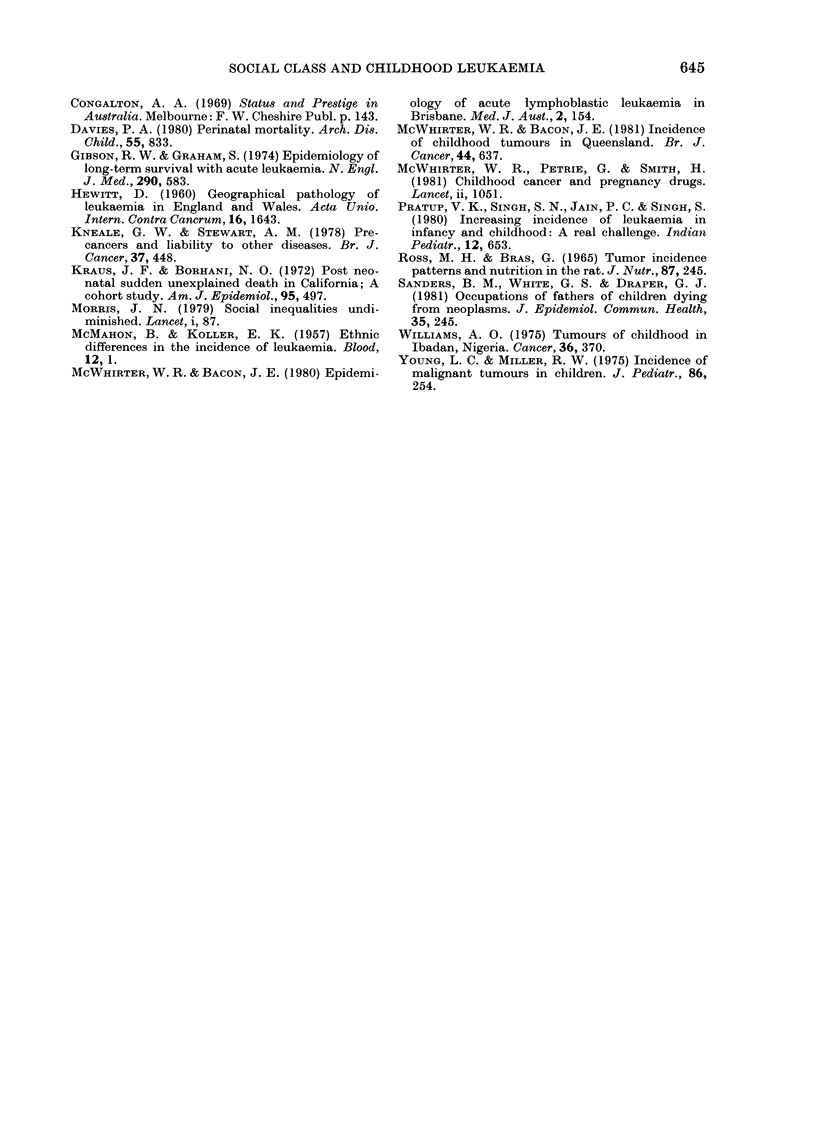# The relationship of incidence of childhood lymphoblastic leukaemia to social class.

**DOI:** 10.1038/bjc.1982.249

**Published:** 1982-10

**Authors:** W. R. McWhirter

## Abstract

A study has been made of the relationship between socio-economic factors and the incidence of acute lymphoblastic leukaemia (ALL) of childhood. It was found that the incidence of childhood ALL in 12 areas of Queensland. Australia, correlated well with some indicators of above-average socio-economic status for these areas. A similar result was found when Brisbane City was studied separately. Social class was determined from the fathers' occupations at the time of diagnosis. There was found to be a higher than expected number of ALL cases in each of the upper 5 social classes and a lower than expected number in the remaining 2 lower classes. Factors associated with differences in lifestyle amongst the various social classes may increase or decrease the risk of development of ALL.


*Br. J. Cancer* (1982) **46**, 640

### THE RELATIONSHIP OF INCIDENCE OF CHILDHOOD LYMPHOBLASTIC LEUKAEMIA TO SOCIAL CLASS

W. R. McWHIRTER

*From the Department of Child Health, University of Queensland, Herston Road, Herston, Queensland* 4029, *Australia*

Received 15 February 1982 Accepted 17 June 1982

**Summary.—A study has been made of the relationship between socio-economic factors and the incidence of acute lymphoblastic leukaemia (ALL) of childhood. It was found that the incidence of childhood ALL in 12 areas of Queensland, Australia, correlated well with some indicators of above-average socio-economic status for these areas. A similar result was found when Brisbane City was studied separately. Social class was determined from the fathers' occupations at the time of diagnosis. There was found to be a higher than expected number of ALL cases in each of the upper 5 social clases and a lower than expected number in the remaining 2 lower classes. Factors associated with differences in lifestyle amongst the various social classes may increase or decrease the risk of development of ALL.**

LEUKAEMIA is the commonest type of malignant disorder in childhood and although its prognosis has improved considerably in recent years, it remains an important cause of death in children. A number of previous publications on the epidemiology of leukaemia have examined leukaemia at all ages rather than childhood leukaemia alone. The distinction may be an important one, since the relative proportions of the various types of leukaemia are quite different in children as compared with adults. The present study is concerned only with acute lymphoblastic leukaemia (ALL), which accounted for 88% of all leukaemias in childhood in Queensland during a 7-year period.

The data for this paper were gathered as part of an ongoing study of childhood cancer epidemiology by the Queensland Childhood Malignancy Registry. The cases occurred during the period 1973–79, which is centred around the Australian Census of 30 June, 1976. The childhood population (under 15 years of age) of Queensland was 571,965 (Australian Bureau of Statistics, 1976). The area of the state is 1,727,000 sq.km and is one of the largest areas in the world to be covered by a single cancer registry. The childhood population of the capital, Brisbane, is 172,037.

### PATIENTS AND METHODS

The Queensland Childhood Malignancy Registry is a population-based registry covering the whole of Queensland. Most cases are found by checking hospital records and, since only a very limited number of hospitals in Queensland treat childhood leukaemia, this research provides almost all the cases occurring in the state. A small number of cases were found from the records of private practitioners.

The lists of deaths from cancer supplied by the Registrar General are also checked, but no additional cases of leukaemia have been found from this source. At least 97% of cases were ascertained (McWhirter & Bacon, 1981). Most of the information about each case is abstracted from the hospital record, but some additional information has been obtained from the Registrar General and the Department of Maternal and Child Health. The information is entered on cards, coded, and stored on computer for subsequent analysis. All cases were diagnosed on the basis of marrow examination with appropriate cytochemistry.

In order to study the regional variations in the incidence of leukaemia, the number of cases in each of the 11 Statistical Divisions (SDs) of Queensland was determined. The Brisbane SD was further subdivided into the 2 subdivisions of Brisbane City and the balance of the Brisbane SD, making a total of 12 areas within the state, each with a known childhood population.

Demographic data were obtained from the Australian Bureau of Statistics (1976). This included information on the population of each area by age group and also the proportions of the population with tertiary qualifications (a diploma or degree), in various occupation groups, and the proportion of students attending non-government schools (by place of residence). Similar data were obtained from the same source for each of the 153 suburbs of Brisbane City.

### RESULTS

During the 7-year period 1973–79 inclusive, there were 127 cases of ALL in children under the age of 15 years (69 males and 58 females). This gives an age-specific incidence of 3·17 per 100,000 per year. The incidence of ALL in each of the 12 areas of Queensland as defined above was calculated (Table I). The variation in incidence throughout the state does not quite achieve statistical significance ($\chi^2$ test for Poisson homogeneity), perhaps because of the small size of the population in some of the statistical divisions. It has however been shown previously that the incidence in Brisbane City is significantly higher than in the balance of the Brisbane Statistical Division (McWhirter & Bacon, 1980). The incidences of ALL in each of the 12 areas in Queensland was compared with some demographic factors for each of these areas (Table II, Fig. 1). In Fig. 1 each point represents one of the 12 areas. Statistically significant positive correlations were found between the ALL incidence in each area and the proportion of the population with tertiary qualifications, the proportion of the population in professional or technical occupations and the proportion of secondary students attending non-Government schools. A significant negative correlation exists between ALL incidence and the proportion of the population in farming, trade, production-process or labouring occupations. Brisbane City contains 153 suburbs. Using demographic data, again obtained from the Australian Census (Australian Bureau of Statistics, 1976), these were divided into

TABLE I.—*Annual age-specific incidence per* 100,000—0–14 *years inclusive*

| Area | Incidence |
|---|---|
| Brisbane City | 4·2 (51) |
| Balance of Brisbane SD | 2·2 (14) |
| Moreton SD | 2·9 (11) |
| Wide Bay-Burnett SD | 1·7 (5) |
| Darling Downs SD | 3·4 (11) |
| Far West SD | 1·6 (1) |
| Fitzroy SD | 3·0 (8) |
| Central-West SD | — (0) |
| Mackay SD | 1·8 (3) |
| Northern SD | 4·7 (14) |
| Far North SD | 2·3 (6) |
| North-Western SD | 3·1 (3) |

Number of cases in parentheses.

TABLE II.—*Spearman correlation coefficients of acute lymphoblastic leukaemia incidence with prevalence of various demographic factors in each of* 12 *areas of Queensland*

| Demographic feature of population | Age (years) of cases | | | | |
|---|---|---|---|---|---|
| | Under 1 | 1–4 | 5–9 | 10–14 | 0–14 |
| With tertiary qualifications | 0·0928 | 0·7990** | 0·7265** | 0·2611 | 0·8973** |
| In professional or technical occupations | 0·1706 | 0·7439** | 0·7180** | 0·1958 | 0·8182** |
| Secondary students attending non-government schools | 0·4410 | 0·7509** | 0·6935* | 0·5583* | 0·8951** |
| In farming, trade, production-process or labouring occupation | −0·4868 | −0·4491 | −0·6410* | −0·1813 | −0·7203** |

* $P<0·05$.
** $P<0·01$.

Fig. 1.—Correlation of ALL incidence with percentage of secondary students at non-government schools. Each point represents one of the 12 areas within Queensland.

Table III.—*ALL incidence correlation (Brisbane City)*

| Proportion of the population in professional or technical occupations | Annual age-specific incidence of ALL (per 100,000) |
|---|---|
| Less than 8·0% | 2·4 (6) |
| 8·0–15·9% | 3·8 (26) |
| 16·0% or more | 7·2 (19) |

Number of cases in parentheses.

$\chi^2 = 7{\cdot}84, P = 0{\cdot}020$.

Table IV.—*ALL incidence correlation (Brisbane City)*

| Proportion of students at non-government schools | Annual age-specific incidence of ALL (per 100,000) |
|---|---|
| Less than 25·0% | 2·8 (12) |
| 25·0–39·9% | 3·9 (22) |
| 40·0% or more | 8·3 (17) |

Number of cases in parentheses.

$\chi^2 = 10{\cdot}2, P = 0{\cdot}006$.

3 categories on the basis of ech of 2 demographic characteristics: the percentage of the population in professional or technical occupations (Table III), and the percentage of students attending non-government schools (Table IV). The annual age-specific incidence of ALL was also calculated for the group of suburbs in each category. The divisions of the population were chosen before analysis to give a reasonable separation within categories while maintaining adequate population numbers in each division. A trend towards a higher incidence of ALL in upper social class suburbs was noted.

The occupation of the father at the date of presentation with ALL could be determined in 109 of the 127 cases. Two cases in the family of which there was no father were excluded. The fathers were then ranked on a 7-point social class scale (Congalton, 1969) on the basis of their occupation. The Congalton scale is an Australian population-determined scale which ranks occupational prestige, and is the most widely used social-class scale in Australia. The distribution of the employed male population of Queensland over 15 years of age amongst the 7 social classes was also determined from census figures. It has been assumed that family size does not vary with social class. If, however, as in other countries, family size

FIG. 2.—Distribution of social class of fathers of ALL cases and of the employed male population in Queensland.

in Australia tends to be larger in lower social class families, the expected number of cases in Classes 6 and 7 would be even greater. Unfortunately, precise information on this point is not available in Australia. As can be seen in Fig. 2, a higher than expected number of ALL cases was found in each of the upper 5 social classes and a considerably lower than expected incidence in the 2 lower social clases ($\chi^2 = 44 \cdot 0$, $P < 0 \cdot 001$, Bartholomew's test for trend, $P < 0 \cdot 01$) (Barlow *et al.*, 1972). In an unselected series of 209 other childhood malignancies, mainly solid tumours, from the same period (McWhirter & Bacon, 1981) there was no significant difference in the distribution of the social class of the fathers compared with that of the employed male population.

### DISCUSSION

In this study it has been shown that childhood ALL occurs with greater incidence in areas of the state which have some indicators of high socio-economic status. These indicators include an above average proportion of the population in professional and technical occupations, an above average proportion of secondary students attending non-Government schools, and a below average proportion of the population in semi-skilled and unskilled occupations. Because these factors might be dependent upon accessibility to non-Government schools or to certain occupations, the cases residing in Brisbane were analysed separately. It was again found that there was a significant increase in ALL incidence in those suburbs where there was a high proportion of students attending non-Government schools, or a high proportion of the population in professional or technical occupations. Since the same trend is seen within a small area, namely Brisbane City, as in the state as a whole, the variations in the incidence of ALL appear to be attributable to characteristics of the population rather than to geographical factors.

This impression is confirmed by comparing the social class distribution of the fathers of the cases of the male population of Queensland (as determined from 1976 census figures). Although the use of census figures as a control population is not ideal, the significance of the shift towards upper social classes amongst the cases is such that it is unlikely to be due to artifact. Additionally the absence of such a shift amongst the non-leukaemic malignancies reduces the likelihood of bias having been introduced during allocation of the cases to a social class.

The higher incidence of childhood ALL in the Brisbane SD as compared with the surrounding semi-rural area comprising the balance of the Brisbane SD has already been reported (McWhirter & Bacon, 1980). In a study of leukaemia at all ages, it was found that the incidence was higher in the south-east of England as compared with the north-west and this seemed to correlate with a gradient in socio-economic status (Hewitt, 1960). Two other reports, one from England and Wales (Sanders *et al.*, 1981) and one from the U.S.A. (Browning & Gross, 1968) have suggested a higher incidence of childhood leukaemia among upper social class families. The incidence of childhood leukaemia is significantly lower in U.S. Blacks compared with Whites (Young & Miller, 1975), probably because of the difference in socio-economic status in the 2 groups (McMahon & Koller, 1957). In some developing countries such as Papua New Guinea and India, a much lower incidence of childhood leukaemia, particularly lymphoblastic, has been reported (Booth & Amato, 1978; Pratup *et al.*, 1980), although it appears to be rising in both these countries. In Nigeria, leukaemia accounted for only 4·5% of all childhood tumours in a large series (Williams, 1975). In contrast, it has recently been reported from the Manchester Children's Tumour Registry (Birch *et al.*, 1981) that the distribution of social class and socio-economic group of their ALL cases did not differ from that of the population as a whole.

The explanation for the relative increase in incidence of ALL in upper social classes is not clear and several hypotheses are possible. Stewart and her colleagues have suggested that children in a pre-leukaemic phase have an increased tendency to infections and that many children die from these infections, especially pneumonia, before developing overt leukaemia (Kneale & Stewart, 1978). Since perinatal (Davies, 1980) and infant (Morris, 1979) mortality and cot deaths (Kraus & Borhani, 1972) are commoner in lower social class families, it is possible that children in these lower social class families die from other causes such as infections or cot deaths before their leukaemia can be recognized. Similarly, the incidence of leukaemia in developing countries might be a consequence of their high infant mortality from other causes.

Alternatively, there may be a proportion of children born with an unrecognized immune deficiency which would predispose them to both infections and leukaemia. Children from lower social classes might be more likely to die of these infections at an early age.

It is also possible that some unknown factor occurs with greater frequency in upper social class environments or that improved standards of nutrition or general physical health in some way favour the development of leukaemia. It has been shown that, in rats, caloric restriction reduces the incidence of spontaneous tumours (Ross & Bras, 1965).

Further studies are required to confirm our findings in other parts of the world. These should be based on series of cases in children ascertained by incidence rather than by death. Since there may be a difference in survival from leukaemia according to social class (McWhirter *et al.*, 1981; Gibson & Graham, 1974), series which include only patients who have died (Sanders & White, 1981) may contain an excess of children from lower social classes. A case-control study in Queensland is being planned to attempt to define more accurately the factors which influence the incidence of childhood ALL in this state. Such studies could help to elucidate the aetiology of ALL.

This research was supported by a grant from the Queensland Cancer Fund. I am grateful to Dr V. Siskind in the Department of Social and Preventive Medicine, University of Queensland, for advice on the statistical analysis.

### REFERENCES

Australian Bureau of Statistics, 1976 Census: (1976) Characteristics of the population and dwellings in local government areas (2429.0).

Barlow, R. E., Bartholomew, D. J., Bremner, J. M. & Brunk, H. D. (1972) *Statistical Inference Under Order Restrictions*, Chapter 3. London: John Wiley & Sons.

Birch, J. M., Swindell, R., Marsden, H. B. & Morris Jones, P. H. (1981) Childhood leukaemia in north west England 1954–1977: Epidemiology, incidence and survival. *Br. J. Cancer*, **43**, 324.

Booth, K., & Amato, D. (1978) Leukaemia in Papua New Guinea. *Trop. Geogr. Med.*, **30**, 343.

Browning, D. & Gross, S. (1968) Epidemiological studies of acute childhood leukaemia. *Am. J. Dis. Child.*, **116**, 576.

CONGALTON, A. A. (1969) *Status and Prestige in Australia*. Melbourne: F. W. Cheshire Publ. p. 143.

DAVIES, P. A. (1980) Perinatal mortality. *Arch. Dis. Child.*, **55**, 833.

GIBSON, R. W. & GRAHAM, S. (1974) Epidemiology of long-term survival with acute leukaemia. *N. Engl. J. Med.*, **290**, 583.

HEWITT, D. (1960) Geographical pathology of leukaemia in England and Wales. *Acta Unio. Intern. Contra Cancrum*, **16**, 1643.

KNEALE, G. W. & STEWART, A. M. (1978) Precancers and liability to other diseases. *Br. J. Cancer*, **37**, 448.

KRAUS, J. F. & BORHANI, N. O. (1972) Post neonatal sudden unexplained death in California; A cohort study. *Am. J. Epidemiol.*, **95**, 497.

MORRIS, J. N. (1979) Social inequalities undiminished. *Lancet*, i, 87.

MCMAHON, B. & KOLLER, E. K. (1957) Ethnic differences in the incidence of leukaemia. *Blood*, **12**, 1.

MCWHIRTER, W. R. & BACON, J. E. (1980) Epidemiology of acute lymphoblastic leukaemia in Brisbane. *Med. J. Aust.*, **2**, 154.

MCWHIRTER, W. R. & BACON, J. E. (1981) Incidence of childhood tumours in Queensland. *Br. J. Cancer*, **44**, 637.

MCWHIRTER, W. R., PETRIE, G. & SMITH, H. (1981) Childhood cancer and pregnancy drugs. *Lancet*, ii, 1051.

PRATUP, V. K., SINGH, S. N., JAIN, P. C. & SINGH, S. (1980) Increasing incidence of leukaemia in infancy and childhood: A real challenge. *Indian Pediatr.*, **12**, 653.

ROSS, M. H. & BRAS, G. (1965) Tumor incidence patterns and nutrition in the rat. *J. Nutr.*, **87**, 245.

SANDERS, B. M., WHITE, G. S. & DRAPER, G. J. (1981) Occupations of fathers of children dying from neoplasms. *J. Epidemiol. Commun. Health*, **35**, 245.

WILLIAMS, A. O. (1975) Tumours of childhood in Ibadan, Nigeria. *Cancer*, **36**, 370.

YOUNG, L. C. & MILLER, R. W. (1975) Incidence of malignant tumours in children. *J. Pediatr.*, **86**, 254.